# Angiotensin-(1–7) and the G Protein-Coupled Receptor Mas Are Key Players in Renal Inflammation

**DOI:** 10.1371/journal.pone.0005406

**Published:** 2009-04-30

**Authors:** Vanesa Esteban, Silvia Heringer-Walther, Anja Sterner-Kock, Ron de Bruin, Sandra van den Engel, Yong Wang, Sergio Mezzano, Jesus Egido, Heinz-Peter Schultheiss, Marta Ruiz-Ortega, Thomas Walther

**Affiliations:** 1 Cellular Biology in Renal Diseases Laboratory, Fundación Jimenez Diaz, Universidad Autónoma Madrid, Madrid, Spain; 2 Department of Obstetrics, University of Leipzig, Leipzig, Germany; 3 Department of Cardiology, Charité, Campus Benjamin Franklin (CBF), Berlin, Germany; 4 Institute for Veterinary Pathology, Freie Universität, Berlin, Germany; 5 Department of Surgery, Erasmus Medical Center, Rotterdam, The Netherlands; 6 Centre for Biomedical Research, Hull York Medical School, University of Hull, Hull, United Kingdom; 7 Division of Nephrology, School of Medicine, Universidad Austral, Valdivia, Chile; L'Istituto di Biomedicina ed Immunologia Molecolare, Consiglio Nazionale delle Ricerche, Italy

## Abstract

Angiotensin (Ang) II mediates pathophysiologial changes in the kidney. Ang-(1–7) by interacting with the G protein-coupled receptor Mas may also have important biological activities.

In this study, renal deficiency for Mas diminished renal damage in models of renal insufficiency as unilateral ureteral obstruction and ischemia/reperfusion injury while the infusion of Ang-(1–7) to wild-type mice pronounced the pathological outcome by aggravating the inflammatory response. Mas deficiency inhibited NF-κB activation and thus the elevation of inflammation-stimulating cytokines, while Ang-(1–7) infusion had proinflammatory properties in experimental models of renal failure as well as under basal conditions. The Ang-(1–7)-mediated NF-κB activation was Mas dependent but did not involve Ang II receptors. Therefore, the blockade of the NF-κB-activating properties of the receptor Mas could be a new strategy in the therapy of failing kidney.

## Introduction

Patients with chronic kidney diseases (CKD) represent a worldwide problem. The prevalence of CKD in the US population is around 11%, and because of the increase in diabetes, obesity and hypertension an exponential increase is predicted for the next decade [1], [2].

The activation of the renal renin angiotensin system (RAS), characterized by elevated ACE expression and increased local angiotensin (Ang) II production, has been found in many human kidney diseases [3]. Ang II, the main peptide of RAS, is a true cytokine that regulates cell growth, inflammation and fibrosis and therefore contributes to renal damage progression [4]. Blockade of Ang II actions, by ACE inhibitors or AT1 antagonists, is one of the current therapeutic strategies with proven beneficial effects in the treatment of chronic renal diseases [5], [6].

Besides Ang II, other Ang peptides, such as Ang IV [Ang-(3–8)] and Ang-(1–7) may also have important biological activities [7]. Especially, Ang-(1–7) has become an angiotensin of interest in the past few years, since its cardiovascular and baroreflex actions counteract those of Ang II [8]. Studies from our group and others in mice deficient for the G protein-coupled receptor Mas and cell transfection experiments gave evidence that *Mas* codes for a functional Ang-(1–7) receptor [9], [10], that is expressed predominantly in testis and in distinct areas of fore-brain such as the hippocampus and amygdada and, less strongly but detectable, in kidney and heart [11]. First suggestions that the *Mas* gene codes for an Ang II-sensitive receptor [12] have been corrected by findings that alterations in intracellular Ca^2+^-concentrations in *Mas*-transfected cells after Ang II treatment could only be confirmed in cells expressing the Ang II receptor AT1 endogenously [13], [14].

Mice deficient for the *Mas* protooncogene present a sustained long-term potentiation in hippocampal neurons and sex-specific alterations in exploratory behaviour [15] and heart rate and blood pressure variability were observed [16].

## Results

### Disease evolution in *Mas*-deficient mice following unilateral ureteral obstruction

The unilateral ureteral obstruction (UUO) model is characterized by interstitial inflammatory cell infiltration, NF-κB activation, apoptosis and fibrosis [17]. Many studies have demonstrated that Ang II, via AT_1_ or AT_2_ receptors, contributes to renal damage following UUO [18]–[19].

**Figure 1:**
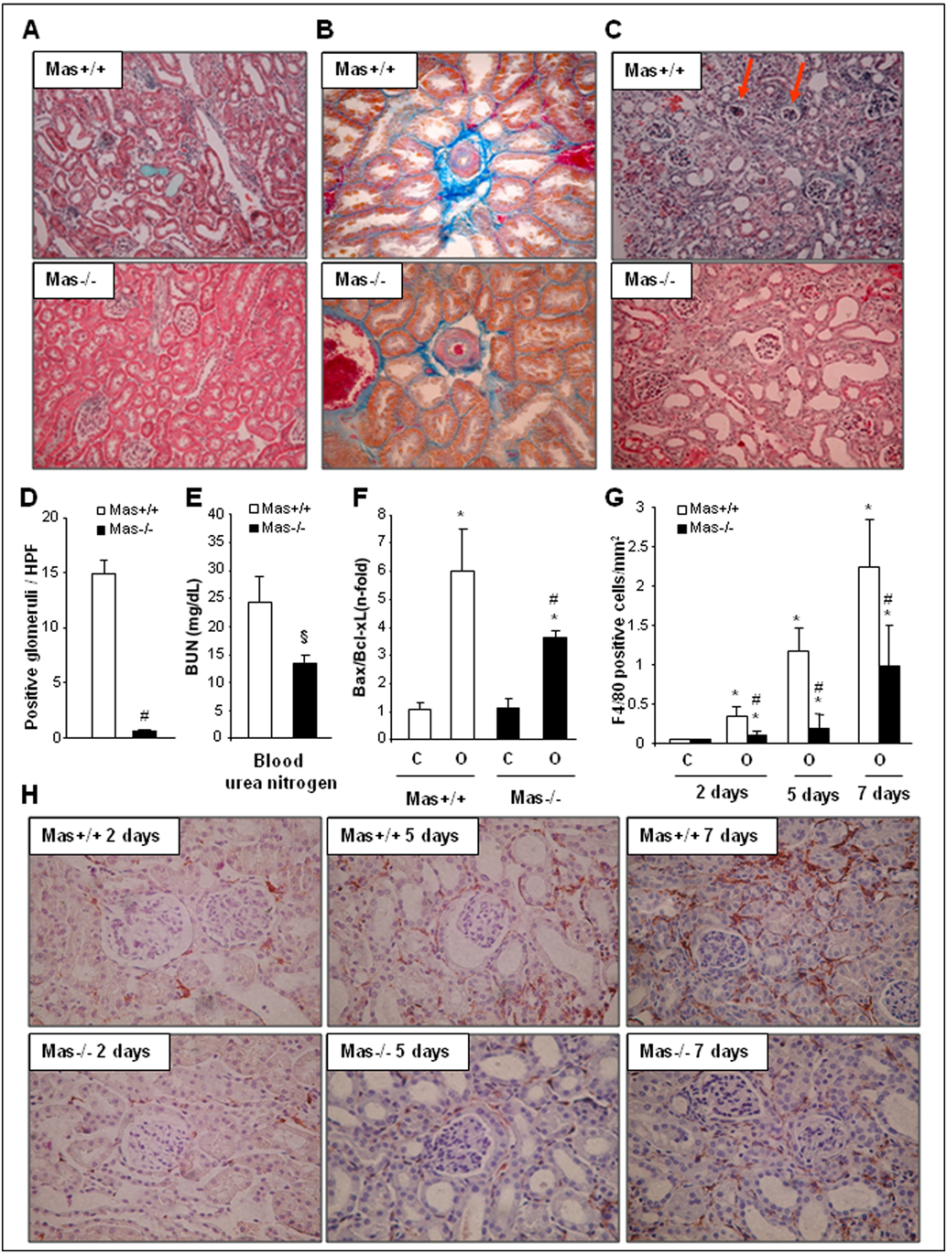
Evolution of renal lesions in the model of unilateral ureteral obstruction (UUO) in *Mas*-deficient and wild-type mice. UUO was set in *Mas*-deficient (Mas−/−) mice and their wild-type controls (Mas+/+), and animals were studied after 2, 5 and 7 days; Masson staining (A) and Azan blue (B) after 2 days and Masson staining after 7 days (C), respectively; magnification: 100×, 200× and 100×, respectively. (D) Shows the quantification of glomeruli with pathomorphological lesions such as collapse, matrix deposition, microthrombi and cellular infiltrates, in UUO kidneys of Mas-deficient mice and their wild-type controls. (E) Demonstrates lower concentrations of blood urea nitrogen in *Mas*-deficient mice 2 days after UUO. (F) Shows evaluation of apoptosis-related proteins at 2 days of UUO. Data of renal Bax/Bcl-xl ratio expressed as n-fold of increase vs. contralateral kidneys of each genotype, expressed as mean±SEM of 6–8 animals per group. Open and black bars represent Mas+/+ or Mas−/−, respectively; C: contralateral, O: obstructed kidneys; * *P*<0.05 vs. contralateral kidney of its own genotype; # *P*<0.05 vs. Mas+/+ obstructed kidney. (G) Shows the computer analysis of monocytes/macrophages scoring 2, 5 and 7 days of UUO. The presence of inflammatory cell infiltration was determined by immunohistochemistry with anti-F4/80 antibody (specific for monocytes/macrophages; brown staining); magnification: 200×. There were no differences between the number of monocytes/macrophages in contralateral kidneys at 2, 5 and 7 days of UUO (data not shown). Results are expressed as F4/80 positive cells/mm^2^ as mean±SEM of 6–10 animals per group. Open bars show data of Mas +/+ and black bars of Mas−/− kidneys, * *P*<0.05 vs. contralateral kidney of the same genotype; # *P*<0.05 vs. Mas+/+ obstructed kidneys; § *P*<0.05 vs. Mas+/+ mice. (H) Illustrates inflammatory cell infiltration in obstructed kidneys (cortex) of Mas+/+ and Mas−/− mice (representative kidneys of 6–10 studied in each genotype, each time point).

As Ang-(1–7) has been shown to have protective cardiovascular effects [21], [22] and this heptapeptide has been also described to have effects in the kidney [23], [24], we investigated effects of deficiency in the Mas receptor associated to Ang-(1–7) signaling in the UUO model. Thus, UUO was performed in Mas-knockout mice (Mas−/−) and their wild-type controls (Mas+/+) and kidneys were studied after 2, 5 and 7 days. The contralateral kidneys of Mas-deficient animals showed no histological alterations and did not differ from contralateral wild-type kidneys. Masson staining of kidney sections two days after UUO showed occasional perivascular mononuclear cell infiltrates and tubular damage in wild-type kidneys, congruent to our previous description [18], while obstructed kidneys lacking Mas showed no inflammatory infiltrates and tubular lesions (**Figure 1A** **, lower panel**). Wild-type UUO kidneys revealed prominent perivascular, interstitial matrix deposition that was less pronounced in Mas−/− mice (**Figure 1B**, blue staining). This difference further increased at the endpoint of 7 days, when wild-type kidneys showed pronounced infiltration of cells in the interstitium and fibrosis while much less stained cells in the knockout kidneys were detectable (**Figure 1C**). Notably, the UUO kidneys with Mas receptors showed occasional glomerular collapse (arrowheads) that was not observed in kidneys lacking the receptor (**Figure 1C**). Glomerular pathology was quantified by counting the glomeruli with pathomorphological lesions such as collapse, matrix deposition, microthrombi and cellular infiltrates. The number of glomeruli with pathomorphological changes was significantly less in *Mas*-deficient UUO kidneys than in kidneys of wild-type mice after 5 days of UUO (**Figure 1D**).

Importantly, the significant less renal damage after UUO in *Mas*-deficient mice led also to a preserved renal function compared to wild-type mice with UUO. Blood urea nitrogen (BUN) (**Figure 1E**) and urea (**data not shown**) were significant less in mice with Mas deficiency two days after UUO. Notably, under sham conditions both strains did not differ in renal function (**data not shown**).

Since these results suggested that the absence of the receptor Mas prevents renal disease progression in the UUO model, we next examined the expression of apoptosis-related proteins that promote (Bax) or protect (Bcl-xL) from cell death. Two days after UUO, the Bax/Bcl-xL ratio increased in the obstructed kidneys of both genotypes in comparison to the contra-lateral control kidneys but significantly less in Mas-deficient mice. This less pronounced increase in the Bax/Bcl-xL ratio in UUO-kidneys of Mas−/− mice implicated induction of less apoptosis (**Figure 1F**).

To dissect the inflammatory response in this model, we examined inflammatory cell infiltration by immunohistochemistry with a specific anti-F4/80 antibody that recognizes murine monocytes/macrophages. In control samples and in non-injured kidneys of both genotypes, only few cells were positive for F4/80 (**data not shown**). Two and five days after UUO, obstructed kidneys of Mas+/+ mice showed scattered infiltrates of mononuclear cells within interstitial spaces focally distributed mainly at juxtamedular level, compared to contralateral kidneys (**Figure 1G** **;** **Figure 1H** **, upper panels**). Obstructed kidneys of Mas-deficient mice lacked this pronounced increase in infiltrating cells (**Figure 1G** **;** **Figure 1H** **, lower panels**). After 7 days of UUO, the presence of monocytes/macrophages infiltrates in Mas-deficient kidneys was significantly lower than in obstructed wild-type kidneys (**Figure 1G** **;** **Figure 1H** **, right panels**). This clearly supports our data that renal Mas deficiency retards UUO damage progression.

### Ang-(1–7) worsens the renal lesions following unilateral ureteral obstruction

**Figure 2:**
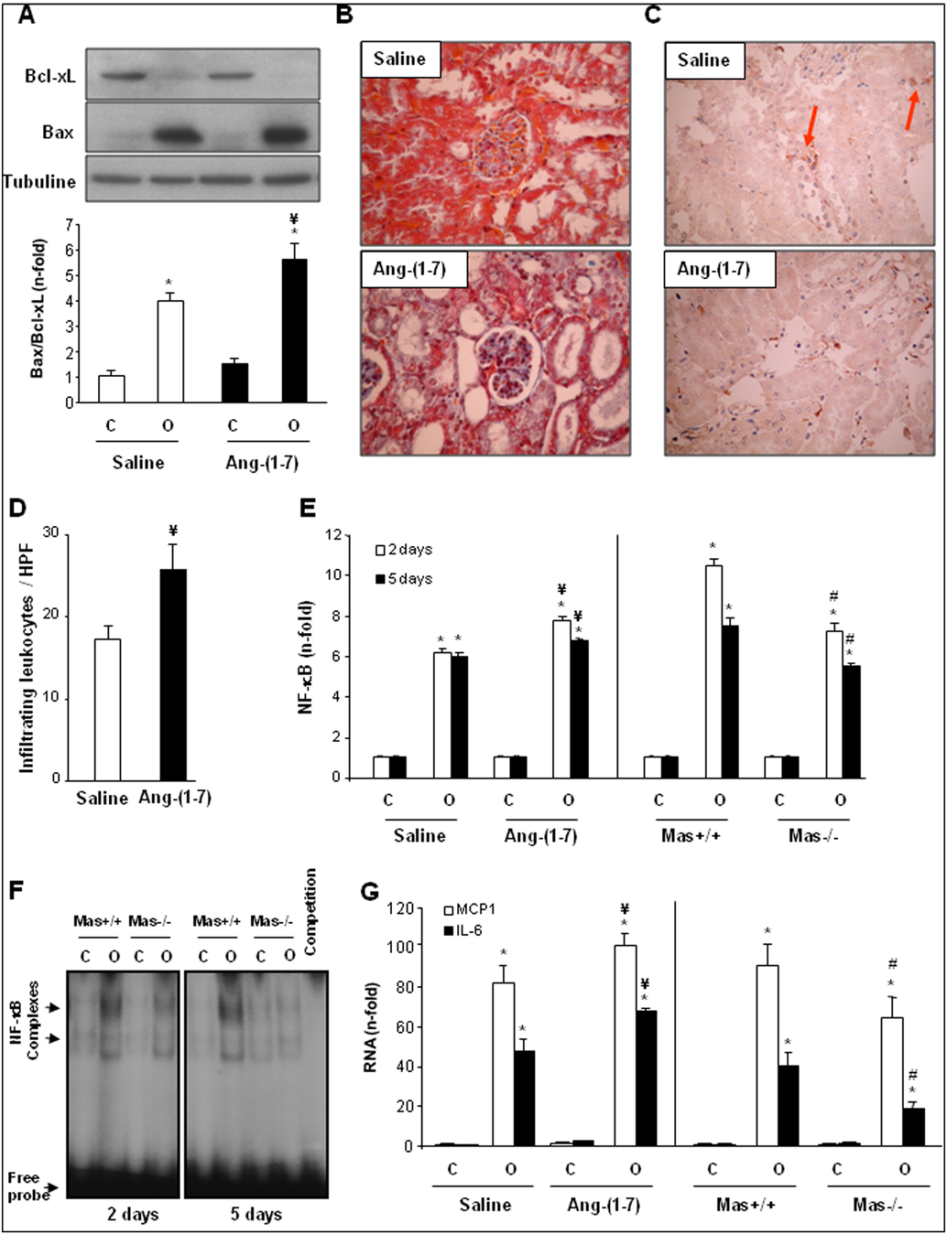
Effect of Ang-(1–7) treatment on apoptosis, fibrosis and inflammatory processes in wild-type mice with unilateral ureteral obstruction (UUO). Wild-type mice underwent UUO and were treated with Ang-(1–7) or saline (systemic infusion by osmotic minipumps). (A) A representative western blot showing renal Bax and Bcl-xL expression (upper panel) and the calculation of the Bax/Bcl-xl ratio (lower panel) after 5 days of UUO. Open and black bars represent data of saline- or Ang-(1–7)-infused mice, respectively, expressed as mean±SEM of 6 animals per group. Figures B and C show representative kidney sections (B: cortex with glomerulus; C: medulla) with van Gieson staining and anti-F4/80 immunostaining, respectively (positive immunoreactivity reveals brown signal [arrowheads]). Magnification: 400×. (D) Infiltrating leukocytes were counted based on their morphology and location within renal cortex, medulla, and pelvis in mice infused with saline or Ang-(1–7). (E) Renal NF-κB activation was measured in wild-type mice infused with saline or Ang-(1–7) and in Mas-deficient mice (Mas−/−) and their respective wild-type controls (Mas+/+) at 2 and 5 days of UUO. The figure shows data of renal NF-κB activity expressed as n-fold increase vs. contralateral kidney as mean±SEM of 6 animals per group analyzed in duplicate. (F) Shows a representative EMSA experiment. Competition assay with a 100-fold excess of unlabeled NF-κB shows the specificity of the binding (marked by arrows). The position of free oligonucleotides is indicated. (G) Shows data of gene expression of proinflammatory factors (MCP-1 and IL-6) obtained by real-time PCR experiments and expressed as n-fold increase vs. contralateral kidney as mean±SEM of 8–10 animals per group; C: contralateral, O: obstructed kidneys. * *P*<0.05 vs. contralateral kidney of the same genotype; # *P*<0.05 vs. Mas+/+ obstructed kidney; ¥ *P*<0.05 vs. saline-infused obstructed kidneys.

If the beneficial effects of Mas deficiency were related to the lacking interaction of Ang-(1–7) with its signaling-associated receptor, detrimental effects of the heptapeptide via Mas on the UUO-initiated pathology in wild-type mice had to be anticipated. Therefore, UUO was performed in two groups of wild-type mice, one receiving Ang-(1–7) through a subcutaneously placed minipump, the other one saline. While Mas deficiency reduced the induction of changes in the ratio of pro-/anti-apoptotic proteins in obstructed kidneys (**Figure 1F**), the infusion of Ang-(1–7) significantly pronounced apoptotic processes (**Figure 2A**). This detrimental effect was already manifest at the second day post surgery and even more significant after 5 days.

Histological evaluation identified in UUO kidneys of Ang-(1–7)-treated wild-type mice an increase in matrix deposition in the mesangial area (blue staining) in comparison to obstructed kidneys of the saline-treated animals (**Figure 2B**). The number of glomeruli with pathomorphological changes was significantly more in UUO kidneys of mice treated with Ang-(1–7) than in kidneys of saline-treated animals after 5 days of UUO (UUO wild-type saline: 5.7±0.8 glomeruli with pathomorphological changes per HPF and UUO wild-type Ang-(1–7): 9.9±2.0 glomeruli with pathomorphological changes per HPF; *P*<0.05).

To investigate whether the more severe phenotype related to an Ang-(1–7) infusion led also to more pronounced inflammation, F4/80-positive cells were stained (exemplarily shown for the medulla in **Figure 2C**). The saline group showed only occasional infiltration of inflammatory cells within interstitial areas (arrowheads). However, there was a marked increase of mononuclear cells in interstitial area in the Ang-(1–7)-infused group implicating more inflammation. This pronounced inflammation was also supported by quantitative analysis counting infiltrating leukocytes. The UUO kidneys of mice that received Ang-(1–7) showed significantly more leukocyte infiltration than the UUO kidneys from the saline treated animals (**Figure 2D**).

To follow the hypothesis that Mas deficiency prevents, and Ang-(1–7) stimulates inflammation we next checked for NF-κB activation. UUO significantly increased renal NF-κB activity in saline-treated and Mas-wildtype mice (obstructed vs. contralateral kidney). Confirming our immunohistological data, this activation was even more pronounced by Ang-(1–7) treatment but significantly reduced in mice lacking the Mas receptor (**Figure 2E and F**).

NF-κB is a pivotal transcription factor in chronic immune and inflammatory diseases [25], [26]. In human kidney diseases elevated renal NF-κB activity correlates with upregulation of proinflammatory parameters [3], [25]–[27]. Although there are no current data in humans, many experimental models have shown that NF-κB blockade (by different maneuvers, including IκB overexpression, NF-κB decoy oligonucleotides, NF-κB inhibitors, ACE inhibition, statins, glucocorticoids and antioxidants) attenuates renal inflammation [4], [18], [26].

We recently described for the UUO model that two different NF-κB inhibitors, PDTC and parthenolide, diminished the inflammatory cell infiltration and downregulated gene expression of several proinflammatory factors [18]. Thus, we further investigated whether NF-κB downstream signaling was also influenced by alterations in the Ang-(1–7)/Mas axis. Obstructed kidneys of wildtype mice showed increased mRNA expression of proinflammatory cytokines (TNF-α, IL-6), and chemokines (MCP-1) compared to the contralateral ones (**Figure 2G**) as previously described [18]. Ang-(1–7) infusion further increased IL-6 and MCP-1 mRNA concentrations, while in Mas-deficient mice, obstructed kidneys presented significant less gene activation compared to Mas-wildtype mice, both at 2 and 5 days (**Figure 2G**). Thus, the combination of results taken from immunohistochemistry and molecular biological techniques consistently proves that Ang-(1–7) is a proinflammatory peptide whose actions are abrogated in the absence of the receptor Mas.

### Impact of Mas and Ang-(1–7) on renal ischemia/reperfusion injury

Following these results in the UUO model, we set out to investigate whether the effects on NF-κB regulation by Ang-(1–7) and Mas are restricted to the UUO model or also act in other models of renal inflammation. We thus introduced a second experimental kidney model that is characterized by an inflammatory component. Since renal ischemia is a major cause of acute and end-stage renal failure [28], producing serious morbidity and mortality, renal ischemia/reperfusion (I/R) injury was chosen to evaluate whether Ang-(1–7) and Mas was involved in the primary mechanisms determining ischemia-mediated renal failure.

**Figure 3:**
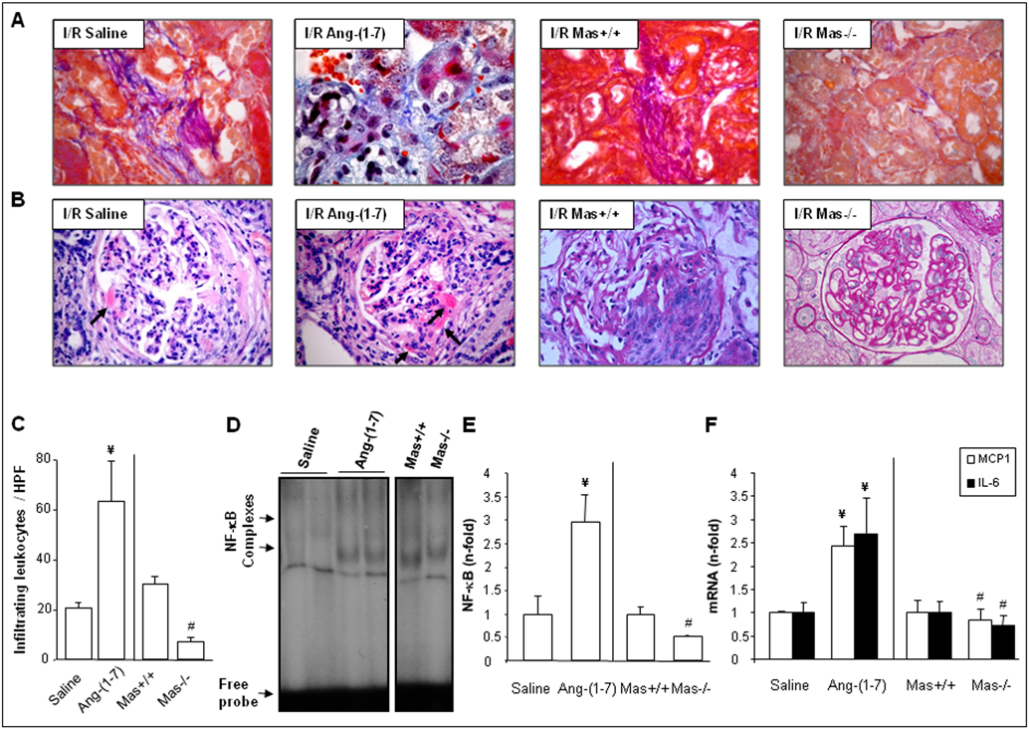
Impact of Mas and Ang-(1–7) on renal lesions in the model of ischemia/reperfusion (I/R). I/R was performed in wild-type mice infused with saline (I/R Saline) or Ang-(1–7) (I/R Ang-(1–7)) and in mice deficient for Mas (I/R Mas−/−) and their wild-type controls (I/R Mas+/+). Animals were studied 3 days after I/R. Figures A and B show Ladewig and PAS staining respectively of group-representing renal sections. Magnification: 400×. (C) Infiltrating leukocytes were counted based on their morphology and location within renal cortex, medulla, and pelvis in mice infused with saline or Ang-(1–7) and in wild-type (Mas+/+) and Mas-deficient mice (Mas−/−). Renal NF-κB activity after I/R was measured by EMSA (D) and calculated (E) as renal NF-κB activity expressed as n-fold increase vs. wild-type controls and shown as mean±SEM of 6–10 animals per group analyzed in duplicate. ¥ *P*<0.05 vs. saline-infused mice; # *P*<0.05 vs. Mas+/+ mice. The right diagram (F) shows mRNA levels of MCP-1 and IL-6 expressed as n-fold increase vs. wild-type controls and expressed as mean±SEM of 6–8 animals per group analyzed in duplicate. ¥ *P*<0.05 vs. saline-infused mice; # *P*<0.05 vs. Mas+/+ mice.

In wild-type animals (I/R saline and I/R Mas+/+), renal ischemia/reperfusion led to diffuse matrix deposition as well as partial glomerular collapse 3 days after reperfusion (**Figure 3A and B**, first and third panels). Furthermore, reduced glomerular perfusion was detected (**Figure 3B**, first and third panels). Compared to these findings, I/R kidneys of mice infused with Ang-(1–7) showed more dramatic tissue destruction as characterized by marked infiltrates of mixed inflammatory cells and more severe diffuse matrix deposition (**Figure 3A and B**, second panel). These kidneys were also characterized by intraglomerular protein precipitates (arrowheads), increased cellularity, and more reduced glomerular perfusion in glomerular capillaries (**Figure 3B**, second panel). In contrast to the more severe pathomorphological changes in the I/R-Ang-(1–7) group, Mas-deficient kidneys showed no evidence of matrix deposition in the renal parenchyma 3 days post ischemia. Notably, there were also significant less structural changes in the glomeruli (**Figure 3A and B**, forth panel). The protective effect of Mas deficiency was also illustrated by the number of glomeruli with pathomorphological changes. The number of altered glomeruli was significantly more in I/R kidneys of mice treated with Ang-(1–7) than in kidneys of saline-treated animals after 3 days of I/R (I/R wild-type saline: 2.8±0.3 glomeruli with pathomorphological changes per HPF and I/R wild-type Ang-(1–7): 4.3±0.4 glomeruli with pathomorphological changes per HPF; *P*<0.01), whereas it was less in Mas-deficient kidneys compared to their wild-type (I/R wild-type: 4.3±0.4 glomeruli with pathomorphological changes per HPF and I/R knockout: 2.3±0.3 glomeruli with pathomorphological changes per HPF; *P*<0.001).

Next, the number of infiltrating leukocytes has been calculated. In I/R kidneys from Ang-(1–7)-treated mice the number of infiltrating leukocytes was significantly more than counted in I/R kidneys from wild-type mice treated with saline (Figure 3C). In contrast, in kidneys from Mas-deficient mice the number of infiltrating leukocytes was less than one quarter of that counted in I/R kidneys from their wild-type controls (**Figure 3C**).

As in the UUO model, Ang-(1–7) infusion increased renal NF-κB activation in comparison to the I/R kidneys of saline-infused animals (**Figure 3D and E**). This was paralleled by significant higher mRNA levels of the proinflammatory cytokines MCP1 and IL-6 (**Figure 3F**). In contrast, the strong beneficial impact of the lack of the Mas receptor on I/R-induced pathomorphological changes was congruent with less NF-κB activation and significant lower renal mRNA of cytokines with proinflammatory properties (**Figure 3D–F**).

### Systemic infusion of Ang-(1–7) into healthy mice induces renal inflammation requiring the Mas receptor

**Figure 4:**
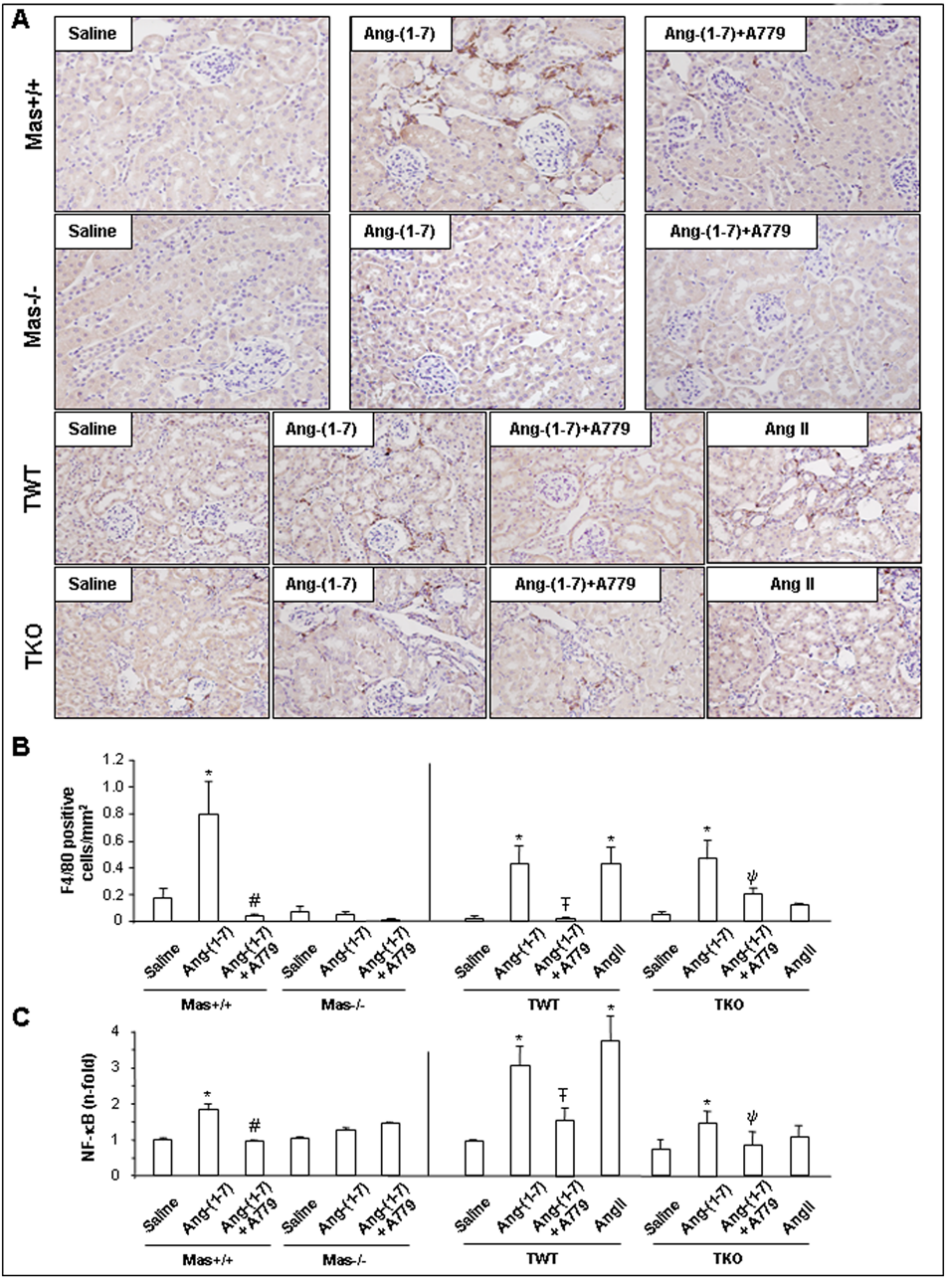
Angiotensin-(1–7) induces inflammatory cell infiltration under physiological conditions. *Mas*-deficient mice (Mas−/−) and mice deficient in all three Ang II receptors (AT1A, AT1B and AT2 = TKO) and their wild-type controls (Mas+/+ and TWT) were infused with Ang-(1–7) or saline for 5 days. Part of the Ang-(1–7)-infused mice were co-treated with the specific Ang-(1–7)-antagonist A779. A shows representative kidney slices stained by anti F4/80 antibody of each group and B summarizes the computer analysis of the monocytes/macrophages score. Results are expressed as F4/80 positive cells/mm^2^ as mean±SEM of 6–10 animals per group. (C) NF-κB data of densitometric analysis of EMSA experiments expressed as mean±SEM of 6–10 animals per group analyzed in duplicate were performed in all 4 investigated genotypes under the three different treatment regimes. *P<0.05 vs. saline-infused of each genotype, # *P*<0.05 vs Ang-(1–7)-infused Mas+/+. Ŧ *P*<0.05 vs. Ang-(1–7)-infused TWT. ψ *P*<0.05 vs. Ang-(1–7)-infused TKO.

To evaluate whether the proinflammatory effects of Ang-(1–7) are depending on a predisposition in failing kidney due to the pathophysiological stimulus or whether elevated circulating Ang-(1–7) concentrations induce renal inflammation already under basal conditions, we administered the heptapeptide to healthy wild-type mice. Systemic minipump infusion of Ang-(1–7) into normal control mice for 5 days caused an important increase in the number of monocytes/macrophages in the interstitium, distributed in a focal manner (**Figure 4A**, upper row). This heptapeptide-mediated increase was completely blocked by co-infusion with A779, an Ang-(1–7) receptor antagonist (**Figure 4A**, upper row), or in Mas-deficient mice. In kidneys of the latter one there were no infiltrating cells detectable, showing similar staining than in saline-infused mice (**Figure 4A**, second row). These results clearly demonstrate the proinflammatory properties of Ang-(1–7) under physiological conditions and that the presence of inflammatory cells in kidneys of animals infused with Ang-(1–7) requires the Mas receptor (**Figure 4B**).

Since there is controversy on the impact of the Ang II receptors AT1 (in rodents two isoforms: AT1A and AT1B) and AT2 on Ang-(1–7)-mediated signaling [7], [8], we used recently generated mice deficient in all three Ang II receptors (triple knockout, TKO) and their wild-type controls (triple wildtype, TWT) [29] to evaluate whether the proinflammatory properties of Ang-(1–7) depend on the expression of Ang II receptors. In both, TKO and TWT, infusion of Ang-(1–7) induces the presence of inflammatory cells, without differences between genotypes, showing that this effect is AT1 and AT2 independent (**Figure 4A**, third and lower row, **Figure 4B**). Notably, the observed effect that A779 treatment significantly diminishes inflammatory cells in Ang-(1–7)-infused wild-type mice was still preserved in mice deficient for all three Ang II receptors.

### Activation of renal NF-κB by angiotensin-(1–7) infusion requires the Mas receptor

To link the increased staining of F4/80-positive cells after Ang-(1–7) infusion to molecular mechanisms, NF-κB activation was detected in the kidneys of the investigated groups. Systemic infusion of Ang-(1–7) for 5 days increased NF-κB DNA-binding activity in the kidney of wild-type mice (Mas+/+ and TWT) (**Figure 4C**) and stimulated release of proinflammatory cytokines (**data not shown**). Congruent with the immunohistochemical findings, there was no increase in renal NF-κB activity in *Mas* knockout mice infused with Ang-(1–7). Pharmacological blockade of Mas by treatment with A779 significantly diminished renal NF-κB activation in response to Ang-(1–7) infusion in wild-type strains (**Figure 4C**). Together, our findings provide robust proof that Ang-(1–7) *in vivo* under non-pathological circumstances initiates renal inflammation by activating the NF-κB pathway. This activation by Ang-(1–7) requires Mas but does not depend on any of the Ang II receptors.

### Ang-(1–7) activates the NF-κB pathway in cultured tubulo-epithelial cells

**Figure 5:**
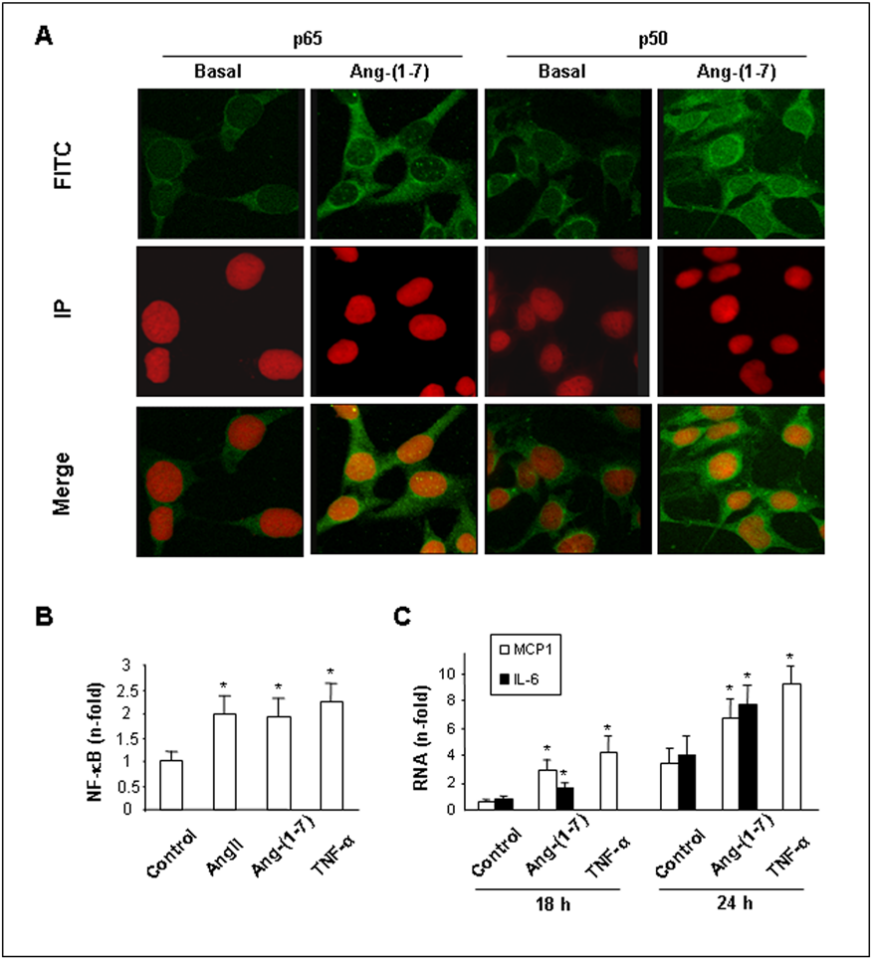
Ang-(1–7) induces nuclear localization of p65 and p50 and proinflammatory cytokines in cultured mouse tubuloepithelial cells. (A) Growth arrested cells were treated with 10^−7^ mol/L Ang-(1–7) and AngII for 1 hour and p65 and p50 subunits were detected by an indirect immunostaining using a mouse FITC-labeled secondary antibody (green staining). Nuclei were stained with propidium iodure (in red). In the merge, the yellow staining shows nuclear localization of the NF-κB proteins after Ang-(1–7) stimulation. The results are representative of 3 independent observations done by confocal microscopy. (B) Growth arrested cells were incubated with 10^−7^ mol/L Ang-(1–7), AngII or TNFα for 1 hour. Thereafter, nuclear extracts were isolated and NF-κB activity was determined by binding assay with a labeled NF-κB oligonucleotide and analyzed by EMSA. Data are expressed as an n-fold increase vs. control cells, as mean±SEM of 4 experiments. *P<0.05 vs. control. (C) Growth arrested cells were stimulated with 10^−7^ mol/L Ang-(1–7) or TNFα for 18 and 24 hours. Gene expression of MCP-1 and IL-6 was analyzed by real-time PCR. Results are expressed as mean±SEM of 4 experiments. *P<0.05 vs. control.

We performed additional *in vitro* studies to visualize the postulated proinflammatory pathway stimulated by Ang-(1–7). At first we visualized the stimulation of NF-κB translocation to the nucleus in cultured mouse tubuloepithelial cells. In control cells a diffuse cytoplasmic immunofluorescence was seen with antibodies against the p65 or p50 subunits of NF-κB (**Figure 5A**). Treatment with 10^−7^ mol/L Ang-(1–7) for one hour led to an intense nuclear fluorescence with both antibodies, proofing nuclear translocation of NF-κB independent of the EMSA technique we used in renal tissue (**Figures 2D** **,** **3C** **and** **4C**). Importantly, the EMSA technique confirmed the immunofluorescence data in growth-arrested tubuloepithelial cells. Ang-(1–7) augmented NF-κB DNA-binding activity after 30 min being maximal with 10^−7^ mol/L after 1 h (*P*<0.05, n = 4) (**Figure 5B**) and diminished to control levels at 90 min (**data not shown**). Notably, NF-κB activation by Ang-(1–7) was blocked in the presence of A779 (**data not shown**).

To better quantify the potency of Ang-(1–7)-mediated NF-κB activation, we compared it to the well-known and powerful NF-κB-activating ability of Ang II and TNFα [26]. Augmentation of NF-κB DNA-binding activity by Ang-(1–7) was in the same range as that for the two very potent proinflammatory factors (**Figure 5B**), illustrating a possible importance of the heptapeptide-mediated stimulation of inflammatory processes in renal diseases.

To evaluate whether Ang-(1–7) also regulates proinflammatory factors under NF-κB-control under *in vitro* conditions, and is also here comparably potent as TNFα, mRNA of proinflammatory factors was quantified. Ang-(1–7) upregulated gene expression of the cytokine IL-6 and the chemokine MCP-1 at both 18 and 24 h (**Figure 5C**) and this stimulation was comparable to the one induced by TNFα.

## Discussion

Over the last decade, evidence accumulated that Ang-(1–7) has cardiovascular protective effects [8], [21], [22] and counteracts detrimental effects of Ang II under pathophysiological conditions [30]. These effects may relate to the heptapeptide's ability of vasorelaxation post myocardial infarction and its blood pressure-lowing effects under hypertensive conditions. However, all data presented here identify Ang-(1–7) and Mas to have also significant impact on renal inflammation and thus may have decisive properties in the pathogenesis and progression of renal failure. Their proinflammatory properties, being equally potent as the stimulatory capacity of Ang II and TNFα, may counteract the common idea of Ang-(1–7) agonists as a therapy concept in cardiac failure.

Nevertheless Ang-(1–7) and Mas' ability to stimulate inflammatory properties in the kidneys are undoubtedly from the data presented here. Although there were first indications for Ang-(1–7) to have such properties, because the peptide can increase the number of white blood cells [31], [32], we were interested in discovering more detailed mechanisms Mas and the heptapeptide may influence inflammatory processes. The recruitment of immune cells into the damaged kidney is a main feature of many renal diseases. These infiltrating inflammatory cells (monocytes/macrophages, T cells, and neutrophils) mediate the initiation and progression of damage by direct cytotoxicity, the secretion of soluble factors, such as proinflammatory cytokines, metalloproteinases, and growth factors, which modulate the local response and increase inflammation within the damage of kidney [33]. However, regulation renal inflammation is complex, involving activation of transcription factors and induction of chemokines and other proinflammatory mediators [25], [34]. Our data implicates that the proinflammatory properties of Ang-(1–7) and the Mas receptor in the kidney are primarily stimulated by a local activation of the NF-κB pathway, and the upregulation of proinflammatory genes under its control, like MCP-1 and IL-6, rather than by systemic effects as stimulating bone marrow-based progenitors of inflammatory cells. This conclusion is also supported by direct stimulating effects of the heptapeptide on cultured renal cells to stimulate the activation of NF-κB and related genes.

Importantly, Ang-(1–7) stimulates proinflammatory factors independently from Ang II receptors. Furthermore, the absence of renal Mas significantly brakes their stimulation of NF-κB and downstream factors and blunts the stimulatory effects of Ang-(1–7). While some experimental studies based on the interference with chemokine expression or inhibition of NF-κB have shown beneficial effects [18], [35], under certain circumstances, this may worsen the disease [36]. For this reason other targets are needed for the treatment of renal diseases. Since Ang-(1–7) promotes and Mas deficiency inhibits disease progression in two independent experimental models, both are candidates for pharmacological intervention.

As recent studies have demonstrated that several chronic kidney diseases, including diabetic nephropathy can be considered as inflammatory diseases [27], [37], our data may open a new avenue in the understanding of the genesis and disease progression due to the identification of two new key players in renal inflammation. As our data identify the lack/blockade of signaling of Mas as preventing renal inflammation, it may point to unique therapy strategies targeting the receptor and thus identified a promising new tool in preventing or delaying inflammatory renal diseases.

## Materials and Methods

### Animals

Three-month old *Mas*-deficient mice [15] and their wild-type controls, both on a C57Bl/6 background, were used in the experiments. Independent sets of C57Bl/6 mice at the same age were used for the infusion experiments (saline or Ang-(1–7). Furthermore, mice deficient in all three Ang II receptors (AT1A, AT1B, AT2; triple knockouts) [29] and their own wild-type controls were used from the breeding colony of Thomas Walther at the FEM, Berlin, Germany. All animals were maintained under standardized conditions with an artificial 12-h dark-light cycle, with free access to food and water. Animals were killed by cervical dislocation, and kidneys were immediately removed and further processed for histological studies or frozen in liquid nitrogen for evaluation of RNA, protein and transcription factor activity. Control (saline-infused) animals and control animals without surgery (sham) of the same age were used as internal controls in all experimental settings. All animal studies were performed according to national guidelines and approved by the institutional animal care committees in Spain and Germany. This research was in compliance with the Guide for the Care and Use of Laboratory Animals published by the OPRR (Office for Protection against Research Risks) of the US National Institutes of Health, Washington, D.C. (NIH Publication No. 85-23, revised 1985).

### Unilateral ureteral obstruction (UUO)

*Mas*-deficient mice (Mas−/−) and their age-matched wild-type controls (Mas+/+) underwent UUO. Mice were anesthetized by pentobarbital injection. The left ureter was ligated with silk (4/0) at two locations and cut between ligatures (obstructed kidney), as described before [18]. Contralateral kidneys served as controls. Animals were studied for 2, 5 and 7 days (n = 6–10 animals per genotype, per time point). Renal function was characterized as described before by others [38].

### Renal ischemia/reperfusion (I/R)

Mice were anesthetized by isoflurane inhalation. Anesthesia was maintained using a mixture of N_2_O/O_2_/isoflurane. Normal body temperature was maintained by placing the animals on heating pads until recovery from anesthesia. Following a midline abdominal incision, the left renal pedicle was localized and the renal artery and vein were dissected. An atraumatic micro-vascular clamp was placed, and the left kidney was occluded during 25 minutes. After inspection for signs of ischemia, the wound was covered with PBS soaked cotton and the animal was covered with a tin foil insulation sheet. After release of the clamp, restoration of blood-flow was inspected visually and a contra-lateral nephrectomy was performed. The excised right kidney was snap frozen and stored at −80°C for further analysis. The abdominal wound was closed in two layers using 5/0 sutures (B. Braun, Melsungen, Germany). The animals were given 0.5 ml PBS subcutaneously and placed under a heating lamp to recover from surgery.

### Peptide application in UUO and I/R

Immediately following UUO or induction of renal ischemia-reperfusion injury, groups of animals were treated with Ang-(1–7) (15 µg/mouse per day) or saline by continuous subcutaneous infusion using osmotic minipumps (Alza Corp., Palo Alto, CA, USA) that were implanted between the scapula (n = 6–10 animals per group). Since Alzet pumps have a lag time of 2 hours before starting to release the peptide, we placed them in PBS solution 2 hours before implantation.

### Systemic peptide infusion to healthy mice

The *in vivo* effect of Ang-(1–7) in the kidney was evaluated by systemic infusion of Ang-(1–7) (dissolved in saline) into mice (subcutaneously by osmotic minipumps, Alza Corp., Palo Alto, CA, USA), at the dose of 45 µg/mouse per day. *Mas*-deficient (Mas−/−), triple knockouts (TKO) and their wild-type controls (Mas+/+, TWT) underwent infusion for 5 days (n = 6–10 animals per group). In parallel sets, the Ang-(1–7) receptor was pharmacologically blocked using the specific Ang-(1–7) antagonist A779 (60 µg/mouse per day, by subcutaneous osmotic minipumps), starting 24 h before Ang-(1–7) infusion and during all the period of study (n = 6–10 animals per group). Peptides were provided by Bachem (Weil am Rhein, Germany).

### Renal histology and inflammatory cell infiltration

Paraffin sections of mouse tissues were prepared and stained using standard histology procedures, including hematoxylin/eosin (HE), Azan blue, Masson, Ladewig and van Gieson, as we described before [18], [39]. The protocol for periodic acid-Schiff (PAS) staining was adapted from Padi & Chopra [40]. The slides were deparafinized through xylene, and hydrated through graded ethanol. Finally, they have been examined by light microscopy.

Inflammatory cell infiltration was determined by monoclonal antibodies against F4/80 antigen (Serotec, Oxford, UK), present in murine monocytes/macrophages. Briefly, paraffin-embedded sections were rehydrated, their endogenous peroxidase blocked, and incubated for 1 hour at 25°C with 8% bovine serum albumin (BSA)/5% goat serum in phosphate-buffered saline (PBS) to eliminate nonspecific protein binding sites. The slides were then exposed (overnight, 4°C) to the monoclonal F4/80 antibody (dilution 1/50). After removing excess antibody, slides were treated with the corresponding anti-IgG biotinylated-conjugated antibody followed by the avidin-biotin-peroxidase complex (Dako, Dako Diagnósticos S.A, Barcelona, Spain), and 3,3′-diaminobenzidine as chromogen. Some tissue samples were incubated without the primary antibody or unrelated IgG, as negative controls.

**One method used to quantify infiltrating cells was image analysis** using a KZ 300 imaging system 3.0 (Zeiss, Munchen-Hallbergmoos, Germany). Briefly, the percentage of the stained area was calculated as the ratio of stained area and the total field area. For each sample, the mean staining area was obtained by analysis of 10 different fields (×200). The staining score is expressed as F4/80-positive cells/mm^2^. The immunohistochemistry experiments were performed in two kidney sections per experimental animal to obtain a mean score for each of them. In all cases, evaluations were performed by two independent observers in a blinded fashion and the mean score value calculated for each mouse.

### Stereological Analyses

The optical dissector method [41] was used to determine the total number of inflammatory cells per kidney. All estimates were performed using a 100× objective on an Olympus BX-50 microscope (Tokyo, Japan). The images were captured by a JVC TK-C1381EG (JVC, Yokohama, Japan) color video camera coupled to a Pentium PC computer. A software program, DH CASTGRID V1.10 (Olympus, Munich, Germany), was used to superimpose a set of unbiased counting frames on the video image. Fields were selected using a systematic uniform random sampling scheme as previously described [41], [42] using a computer-driven motorized stage (Multicontrol 2000; ITK, Lahnau, Germany). A microcator (Heidenhain D83301; Heidenhain, Traunreut, Germany) was attached to the microscope stage and monitored depth measurement.

The leukocytes were identified based on their morphology and location within the renal cortex, medulla, and pelvis. The frame size for counting leukocytes was 2820 µm^2^. On the other hand, 4 frames with a total of 940 µm^2^ frame size were used to count glomerular injury (collapse, matrix deposition, thrombi). The difference in the number of fields and frame sizes depended on the frequency of the cells observed. Slides were masked prior to each type of quantification (cell number and apoptosis as outlined below) to facilitate unbiased counting.

### Cell Culture

#### A. Conditions

The murine tubuloepithelial cells (MCT cell line) were kindly donated by Dr. E.G. Neilson (Pennsylvania University, Philadelphia, PA, USA). Cells were cultured in petri-dishes with RPMI 1640 medium (BioWhitaker, Verviers, Belgium) supplemented with 10% fetal calf serum (FCS) and penicillin/glutamine (Gibco BRL, Paisley, Scotland, UK).

#### B. Peptide treatments

MCT cells were serum-depleted for 24 h and then stimulated during 18 h or 24 h with AngII or Ang(1–7) 10^−7^ mol/L (Bachem, Weil am Rhein, Germany), or TNF alpha 100 U/ml (Immunogenix corp., Los Angeles, CA, USA) in serum-free medium.

#### C. Immune-fluorescence visualizing NF-κB translocation

Immuno-fluorescence staining for p65 and p50 was done in MCT cells growing in coverslips. After stimulation, cells were washed with PBS, fixed in merckofix (Merck, Darmstadt, Germany) and treated with 0.1% Triton-X 100 for 1 minute on ice to permeabilize nuclear membranes and blocking with 10% sheep serum and 4% BSA in PBS for 1 hour. NF-κB subunits were detected by rabbit polyclonal anti-p65 or p50 antibodies (1∶75 dilution in 4% sheep serum and 1% BSA in PBS; Santa Cruz Biotechnology, Inc, Santa Cruz, CA, U.S.A.) followed by FITC-conjugated goat anti-rabbit IgG antibody (1∶100 dilution in 1% BSA in PBS). Nuclei were stained with propidium iodide (1 µg/ml) for 30 minutes. Coverslips were mounted in mowiol (Calbiochem, Merck KGaA. Biosciences, Darmstadt, Germany) and examined by a laser scanning confocal microscope (Leica Microsistemas, Alcobendas, Spain).

### Quantitative real-time PCR

Total RNA was isolated with Trizol (Gibco BRL, Paisley, Scotland, UK) with subsequent chloroform-isopropanol extraction according to the manufacturer's instructions. Two µg of RNA underwent random primed reverse transcription using a modified Maloney murine leukemia virus reverse transcriptase (Superscript II; Life Technologies, Gaithersburg, MD, USA) for 10 minutes at 25°C and 37°C for 2 hours. Proinflammatory gene expression was analyzed by real-time PCR, performed on an ABI Prism 7500 sequence detection PCR system (Applied Biosystems, Foster City, CA, USA) according to manufacturer's protocol. After an initial hold of 2 minutes at 50°C and 10 minutes at 95°C, the samples were cycled 40 times at 95°C for 15 seconds and 60°C for 60 seconds. For all quantitative cDNA analysis, the **Δ**Ct technique was applied. Assay IDs used were MCP-1, Mm00441242_m1 and IL-6, Mm00446190_m1. To normalize data different approaches were done using several housekeeping genes, including GAPDH, Histone-3 and 18 s ribosomal RNA expression (assay IDs: Mm99999915_g1 and Hs99999901_s). All primers, probes, and reagents were obtained from Applied Biosystems (Foster City, CA, USA). All measurements were performed in duplicate. Controls consisting of ddH_2_O were negative in all runs.

### Determination of NF-κB activity with electrophoretic mobility shift assay (EMSA)

#### A. Protein extraction

For protein extraction from tissues, frozen kidney pieces were pulverized in a metallic chamber and resuspended in a cold extraction buffer [20 mmol/L HEPES-NaOH (pH 7.6), 20% (vol-vol) glycerol, 0.35 mol/L NaCl, 5 mmol/L MgCl_2_, 0.1 mmol/L EDTA, 1 mmol/L DTT, 0.5 mmol/L PMSF]. The homogenate was vigorously shaken for 30 minutes, and the insoluble materials precipitated by centrifugation at 40,000 g for 30 minutes at 4°C. For protein extraction from cultured cells, cells were resuspended in extraction buffer (10 mmol/L HEPES, pH 7.8, 15 mmol/L KCl, 2 mmol/L MgCl_2_, 0.1 mmol/L EDTA, 1 mmol/L dithiothreitol, 1 mmol/L PMSF) and homogenized. Nuclei and cytosolic fractions were separated by centrifugation at 1,000×*g* for 10 minutes. The nuclei were resuspended in extraction buffer to a final concentration of 0.39 mol/L KCl and centrifuged at 100,000×*g* for 30 minutes. Supernatants dialyzed overnight against a binding buffer containing 20 mmol/L HEPES-NaOH (pH 7.6), 20% (v/v) glycerol, 0.1 mmol/L NaCl, 5 mmol/L MgCl_2_, 0.1 mmol/L EDTA, 1 mmol/L dithiothreitol, and 0.5 mmol/L PMSF. The dialysates were cleared by centrifugation at 10,000×*g* for 15 minutes at 4°C and frozen at −80°C. Protein concentration was quantified by the bicinchoninic acid method (Pierce, Rockford, IL, USA).

#### B. Electrophoretic mobility shift assay

NF-κB activity was evaluated by binding of 60 µg of tissue extracts of tissue or 8–10 µg of nuclear extracts from cells, as described [18]. NF-κB consensus oligonucleotide (5′-AGTTGAGGGGACTTTCCCAGGC-3′) was end-labeled with [γ-^32^P]-ATP (Amersham, Buckinghamshire, UK) and T4 polynucleotide kinase (Promega, Madison, WI, USA). Samples were equilibrated for 10 minutes in a binding buffer [4% glycerol, 1 mmol/L MgCl2, 0.5 mmol/L EDTA, 0.5 mmol/L dithiothreitol, 50 mmol/L NaCl, 10 mmol/L Tris-HCl, pH 7.5, and 50 mg/ml of poly(dI-dC)] (Pharmacia LKB, Uppsala, Sweden), then the NF-κB consensus oligonucleotide labeled [γ-^32^P]-ATP (0.35 pmol) was added and incubated for 20 minutes at room temperature.

Negative controls without cellular extracts, and competition assays with a 100-fold excess of unlabeled NF-κB, mutant NF-κB and AP-1 (unrelated) oligonucleotides, were performed to establish the specificity of the reaction (not shown). When competition assays were done, the unlabeled probe was added to this buffer 10 minutes prior to the addition of the labeled probe. The results of EMSA experiments were analyzed using a Densitometer (GS-800, Biorad, Alcobendas, Madrid, Spain). The specificity of the antibodies was confirmed by Western blot. Oligonucleotides were from Promega Corp. (Madison, WI, USA). The reaction was stopped by adding gel-loading buffer (250 mmol/L Tris-HCL, 0.2% bromophenol blue, 0.2% xylene cyanol, and 40% glycerol) and protein-DNA complexes were separated on a nondenaturing, 4% acrylamide gel in Tris-borate. The gels were dried and exposed to X-ray film.

### Western blot analysis

Protein levels were assessed by Western blotting. Total proteins were resolved on 12% sodium dodecyl sulfate-polyacrylamide gels, electrophoretically transferred to polyvinylidene difluoride membranes, blocked (in buffer containing 0.01 mM Tris, pH 7.5, 0.4 M NaCl, 0.1% Tween-20, 1% bovine serum albumin, and 5% milk), and incubated for 18 h at 4°C with Bax and Bcl-xL antibodies (1∶1000 and 1∶500, respectively) (Santa Cruz Biotechnology, Santa Cruz, CA, USA). Detection was performed with peroxidase-conjugated secondary antibody, using an ECL chemiluminescence kit (Amersham, Arlington Heights, IL, USA).

### Statistical analysis

Since all investigated parameters were normally distributed having continuous variables with equal variances, we analyzed with student *t* test or ANOVA. For UUO, all parameters investigated did not differ between contralateral kidneys and kidneys from control mice (without surgery) in each genotype studied (**data not shown**). Data are expressed as mean±SEM. A *P*<0.05 was considered significant. Tests were done using the SPSS 11.5 software package.
